# Slow Voltage Relaxation of Silicon Nanoparticles with a Chemo-Mechanical Core–Shell Model

**DOI:** 10.1021/acsami.4c12976

**Published:** 2024-11-26

**Authors:** Lukas Köbbing, Yannick Kuhn, Birger Horstmann

**Affiliations:** † Institute of Engineering Thermodynamics, German Aerospace Center (DLR), Wilhelm-Runge-Straße 10, Ulm 89081, Germany; ‡ Helmholtz Institute Ulm (HIU), Helmholtzstraße 11, Ulm 89081, Germany; § Faculty of Natural Sciences, Ulm University, Albert-Einstein-Allee 47, Ulm 89081, Germany

**Keywords:** lithium-ion batteries, solid-electrolyte interphase (SEI), silicon anode, voltage relaxation, voltage hysteresis, chemo-mechanical core−shell model, SEI mechanics, Garofalo viscosity

## Abstract

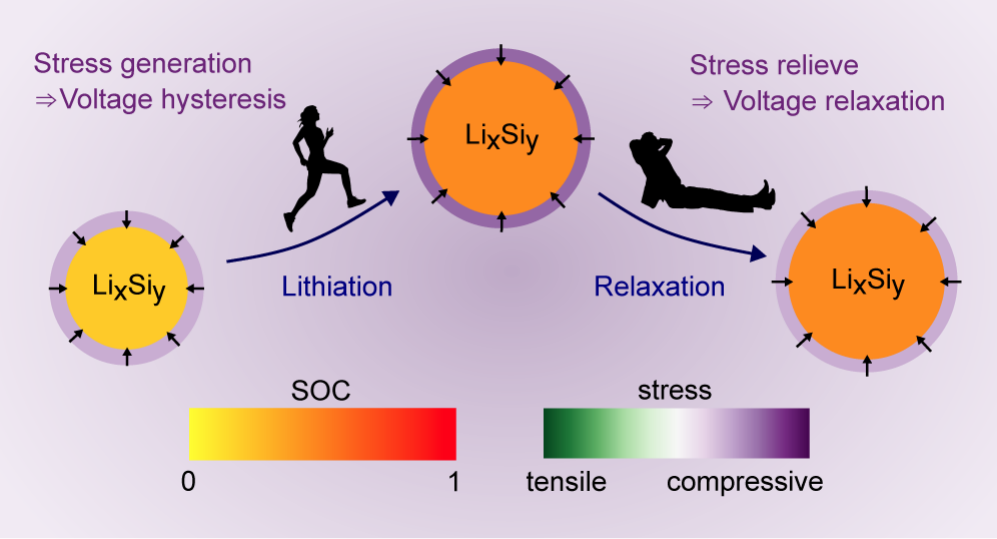

Silicon presents itself as a high-capacity anode material for lithium-ion batteries with a promising future. The high ability for lithiation comes along with massive volume changes and a problematic voltage hysteresis, causing reduced efficiency, detrimental heat generation, and a complicated state-of-charge estimation. During slow cycling, amorphous silicon nanoparticles show a larger voltage hysteresis than after relaxation periods. Interestingly, the voltage relaxes for at least several days, which has not been physically explained so far. We apply a chemo-mechanical continuum model in a core–shell geometry interpreted as a silicon particle covered by the solid-electrolyte interphase to account for the hysteresis phenomena. The silicon core (de)lithiates during every cycle while the covering shell is chemically inactive. The visco-elastoplastic behavior of the shell explains the voltage hysteresis during cycling and after relaxation. We identify a logarithmic voltage relaxation, which fits with the established Garofalo law for viscosity. Our chemo-mechanical model describes the observed voltage hysteresis phenomena and outperforms the empirical Plett model. In addition to our full model, we present a reduced model to allow for easy voltage profile estimations. The presented results support the mechanical explanation of the silicon voltage hysteresis with a core–shell model and encourage further efforts into the investigation of the silicon anode mechanics.

## 1 Introduction

For the enhancement of next-generation lithium-ion batteries, research and industry consider the application of pure silicon anodes.^1−3^ Silicon is a popular choice as it is an abundant and cheap material. Anodes made of silicon possess a high theoretical capacity, leading to a massive volume expansion of up to 300% during lithiation and respective shrinkage during delithiation.^4^ The massive deformations induce significant stresses inside the anode material, causing fracture of large silicon particles above a critical diameter of 150 nm.^5^ Larger silicon particles suffer from cracks, particle pulverization, and are prone to losing contact with the current collector.^6^ Anodes made of silicon nanoparticles promise a higher stability and cycle life compared to anodes with larger silicon particles. Thus, research and industry focus on the behavior of nanostructured silicon anodes.^7^

A severe challenge for the commercialization of silicon anodes is the handling and possible reduction of the voltage hysteresis observed in various experiments.^8−11^ Silicon anodes reveal a different voltage during slow lithiation compared to delithiation, reducing efficiency and causing detrimental heat generation.^12,13^ Experiments observe this hysteresis phenomenon of amorphous silicon anodes in thin-film geometries, micron-sized particles, and nanoparticles.

Literature discusses different reasons for the voltage hysteresis: mechanics and plastic flow of silicon in thin-film geometries,^14,15^ concentration gradients due to slow diffusion in micrometer-sized particles,^16−18^ phase transformation in the very first cycle,^19^ and slow reaction kinetics.^20^ As demonstrated in our previous paper,^21^ these hypotheses are not able to explain the observed voltage hysteresis in GITT experiments with anodes based on amorphous silicon nanoparticles. Therefore, we developed a new chemo-mechanical core–shell model with the plastic flow of the shell,^21^ which explains the observed OCV hysteresis. The enlarged hysteresis during slow cycling is modeled with viscous behavior of the shell. Our previous model can describe the short-term voltage relaxation during GITT measurements for at most 1 h.

Recent experimental results unveil a slow, nonexponential voltage relaxation behavior for at least 300 h, which was so far neither experimentally observed nor theoretically explained on this extended time scale.^11^ Particularly, the observed slow relaxation process once again rules out a diffusional origin with exponential relaxation behavior. Moreover, although the slow voltage relaxation is in line with the mid-term experimental findings of Sethuraman et al.,^20^ their theoretical explanation with reaction kinetics in the Tafel regime requires unreasonable parameter values for the exchange current density and the transfer coefficients. Thus, the novel long-term relaxation measurements strongly support our mechanical explanation. In this article, we propose a viscosity model that fits the experimental results. Our chemo-mechanical consideration as a core–shell model provides a consistent picture of the silicon hysteresis and its dynamics over several time scales.

The core–shell model can be interpreted as a silicon nanoparticle covered by the solid-electrolyte interphase (SEI). The SEI arises on anode particles due to electrolyte decomposition,^22−26^ driven via the electron diffusion mechanism.^27,28^ Moreover, the native silicon oxide layer^29−31^ or artificial coatings^10^ can contribute to the SEI and influence the lithiation behavior of silicon, even for solid electrolytes.^32^ Supporting the impact of the SEI, the inner SEI is reported to be robust^33−35^ and beneficial for the mechanical integrity of the silicon anode.^1,36,37^ This mechanism can also explain the hysteresis of larger silicon particles due to particle pulverization, causing nanoparticles surrounded by freshly formed SEI.^6,38^

An alternative interpretation is the occurrence of active silicon nanodomains in larger silicon particles surrounded by chemically inactive regions. Literature reports the existence of silicon nanodomains for amorphous silicon under high pressure,^39^ for crystalline silicon,^40^ and generically for silicon oxide particles.^41,42^ In general, the presence of nanodomains is independent of the anode geometry.

This manuscript builds on our previous explanation of the voltage hysteresis of silicon nanoparticles by the chemo-mechanical core–shell coupling.^21^ However, this manuscript focuses on the examination and interpretation of the long-term voltage relaxation process of silicon anodes, considering an adequate viscosity model. We explain the basic principles of our chemo-mechanical core–shell model in Section 2. Furthermore, we introduce the Garofalo viscosity model necessary because of the large stresses arising inside the shell and discuss its behavior in the core–shell system with an analytical approximation and a reduced model. In Section 3, we describe the recent experiments performed by Wycisk et al.,^11^ which we analyze in detail in Section 4. In conclusion, we present a consistent description of the observed slow voltage relaxation, hysteresis shape, C-rate dependence, and voltage transition profiles.

## 2 Theory

**Figure 1:**
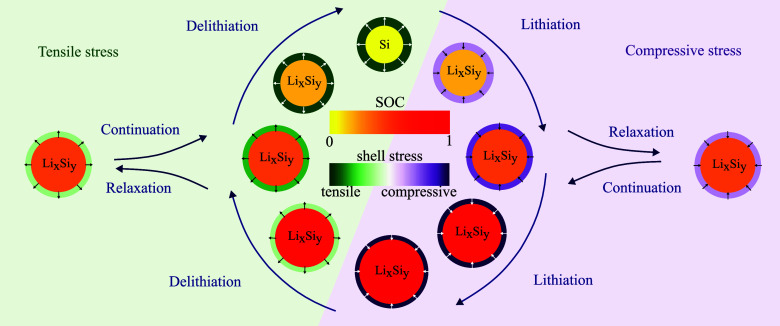
Scheme of volume changes and shell stress during lithiation, delithiation, and relaxation periods.

Our theoretical framework describes the behavior of a core–shell system, where the silicon particle as core can lithiate and delithiate while the shell is chemically inactive and deforms only mechanically as illustrated in Figure 1. We have presented the foundations of the chemo-mechanical core–shell model used in this study in our previous publications.^21,35^ In the following, we summarize the most important assumptions and equations. Further, we highlight advancements compared to our previous works.

### 2.1 Chemo-Mechanical Core–Shell Model

The silicon particle core deforms due to the chemical lithiation and delithiation , elastic deformation , and plastic deformation  when reaching the yield criterion. The large deformation approach determines the total deformation  as
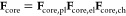
1The concentration of lithium atoms  inside the silicon particle expressed in the undeformed Lagrangian frame determines the chemical deformation
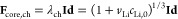
2with *v*_Li_ the molar volume of lithium inside silicon.

The strain tensors  read
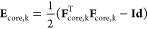
3where the subscript  indicates the kind of deformation, which is either the total deformation or one of the mentioned deformation contributions from eq 1.

The Cauchy stress **σ** describes the stress in the deformed Euler frame and the Piola–Kirchhoff stress  describes the stress in the undeformed Lagrangian frame with . The Piola–Kirchhoff stress due to elastoplastic deformation reads
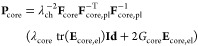
4with the first Lamé constant λ_core_ and the second Lamé constant *G*_core_.

Due to the chemo-mechanical coupling, the stress inside the particle affects the voltage as
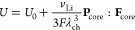
5with the true open-circuit voltage (OCV) of silicon *U*_0_ and the Faraday constant *F*.

The differential equations of interest inside the particle are the continuity equation for the time derivative of the lithium concentration , the momentum balance, and the equation for the plastic flow rate ,
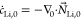
6
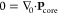
7
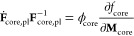
8For the lithiation equation, we define the lithium flux , the electro-chemo-mechanical potential , the mobility , and the diffusion coefficient *D*_Li_. At the particle boundary, the (de)lithiation rate determines the lithium flux . For the plastic flow, the von Mises yield criterion  determines plasticity with  the deviatoric part of the adapted Mandel stress , the reversible deformation , and the yield stress . The consistency condition  determines the plastic multiplier ϕ_core_.

For the shell behavior, we assume that the shell deforms only mechanically, namely elastically and plastically,
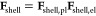
9leading to massive mechanical strains and stresses when experiencing the significant volume change of the silicon particle during cycling. Analogous to the particle, eq 3 determines the strain tensors  inside the shell.

In addition to the elastoplastic stress  determined analogously to eq 4, we consider the viscous behavior of the shell. To describe large viscous stresses during cycling on the one hand and small viscous stresses during relaxation on the other hand, we use the Garofalo law or inverse hyperbolic sine law
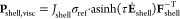
10calculated component-wise and presented initially in ref.^43^. The parameter σ_ref_ describes as a reference stress the magnitude of viscous stress at a certain strain rate. The parameter τ describes the time constant of the system and the dependence on the strain rate. In this study, we use the Garofalo viscosity model stated in eq 10 instead of a standard Newtonian model or a shear-thinning model^21^ to account more adequately for the complexity of the mechanical behavior. The particular functional dependence of the Garofalo law is reasoned in ref.^44^ by a change in the energy landscape due to mechanical deformations and lattice distortions. Furthermore, positive entropy production is guaranteed analogously to the derivation in ref.^21^, as the inverse hyperbolic sine is positive for positive arguments and negative for negative ones.

The differential equations of interest inside the shell are the momentum balance and the equation for plastic flow,
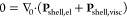
11
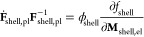
12The yield criterion  is determined by the deviatoric part  of the adapted elastic Mandel stress  and the plastic multiplier ϕ_shell_ results from the consistency condition .

Note that we model the mechanical deformations on a continuum scale. Thus, the visco-elastoplastic behavior is not necessarily an intrinsic property of a single material domain. Instead, interfaces and grain boundaries of multiple crystal domains can determine the continuum mechanics. Hence, the described visco-elastoplasticity can be a consequence of repeated partial cracking and healing, as discussed for the SEI in ref.^35^. This description is reasonable because the literature does not observe significant fracture of the inner SEI layer on silicon.^33−35^

We assume that the surfaces of the silicon core and the shell stick tightly together, meaning that the radial part of the stress coincides
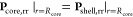
13when evaluated at the core–shell interface *r* = *R*_core_. Due to the merely mechanical deformation of the shell, significant stresses arise inside the shell, impacting the silicon particle stress and voltage.

As presented in ref.^21^, the expansion of the silicon particle during lithiation leads to a mechanical reaction of the shell, namely, first elastic and then plastic deformation. The strains inside the shell generate significant compressive stress acting on the silicon particle as visualized in Figure 1. Additionally, viscous behavior increases the total compressive stress during lithiation depending on the strain rate. During the subsequent delithiation, tensile stress originates from elastic and plastic deformations as well as viscosity, which causes a stress hysteresis inside the shell, impacting the voltage of the silicon particle according to eq 5. Hence, the visco-elastoplastic behavior of the shell describes the voltage hysteresis observed for silicon nanoparticles.

### 2.2 Analytical Approximation for the Voltage Relaxation

To gain an analytical approximation for the voltage relaxation, we investigate the behavior of the presented chemo-mechanical core–shell model in a simplified setup. Thus, we analyze all local variables at the interface accounting for the central role of the interface coupling. In the following, we discuss several assumptions paving the way to a simplified analytical expression.

First, we choose the simplified description that during relaxation the silicon particle behaves purely elastically according to Hooke’s law
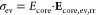
14with Young’s modulus *E*_core_ and the elastic radial strain of the core  due to viscous stress of the shell.

Furthermore, we consider only the viscous stress contribution inside the shell as the elastic stress of the shell stays constant, i.e., 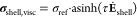
15The time evolution of the radial stress component in the silicon particle resulting from the time derivative of eq 14 states
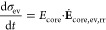
16The silicon core deforms only elastically during relaxation of viscous shell stress and isotropically, thus
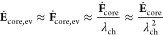
17and
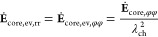
18The radial and tangential stresses are related by the momentum balance as
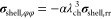
19with the parameter  defined by the core radius *R*_core_ and the shell thickness *L*_shell_.

Using eqs 13, 15, 16 and 19, we find the differential equation for the radial stress component
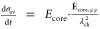
20
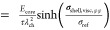
21
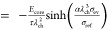
22We solve the simplified differential equation in eq 22 analytically to describe the whole time dependence with a single analytical solution
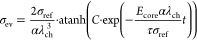
23where the constant *C* can be determined from the boundary condition at time *t* = 0 with .

For the calculation of the stress effect on the silicon voltage according to eq 5, we approximate the deformation of the silicon particle core as purely chemical, , and we assume isotropic stress distribution inside the particle . Therefore, the impact of the stress on the voltage according to eq 5 simplifies to  in the reduced model and the voltage relaxation reads
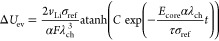
24

To understand the origin and the regimes of the convoluted functional behavior in eq 20, we analyze the relaxation behavior in the limits of large and low stress magnitudes in Section SI. Due to the importance of the long-term relaxation, here we present only the large stress limit. In the limit of large compressive stress, the differential eq 22 simplifies to
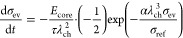
25The analytical solution for this differential equation is
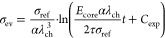
26with the integration constant *C*_exp_ determined from the boundary condition.

Thus, the voltage relaxation according to the Garofalo viscosity
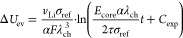
27reveals logarithmic behavior in the large stress limit.

### 2.3 Reduced Model Equations

Complementary to our full model presented in Section 2.1, we derive a reduced model with the key features in Section SII. The reduced model describes the elastic stress contribution of the core at the interface between core and shell due to elastoplastic behavior of the shell σ_ee_ and due to viscous behavior of the shell σ_ev_.

The system of equations defining the reduced chemo-mechanical hysteresis model reads
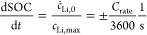
28
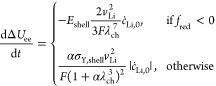
29
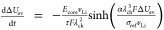
30
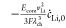
31with the parameter  and the yield condition for plastic flow for the reduced model
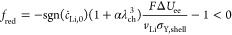
32

The equations defining the reduced model describe the silicon anode voltage as . Eq 28 states the change of SOC for lithiation (+) and delithiation (−). The upper case in eq 29 describes the voltage evolution caused by elastic behavior of the silicon core due to elastic behavior of the shell. The lower case describes elastic core stress due to plastic behavior of the shell. The first term in eq 30 considers the viscous shell stress relaxation. The second term considers viscous shell stress increase because of silicon volume changes.

**Figure 2:**
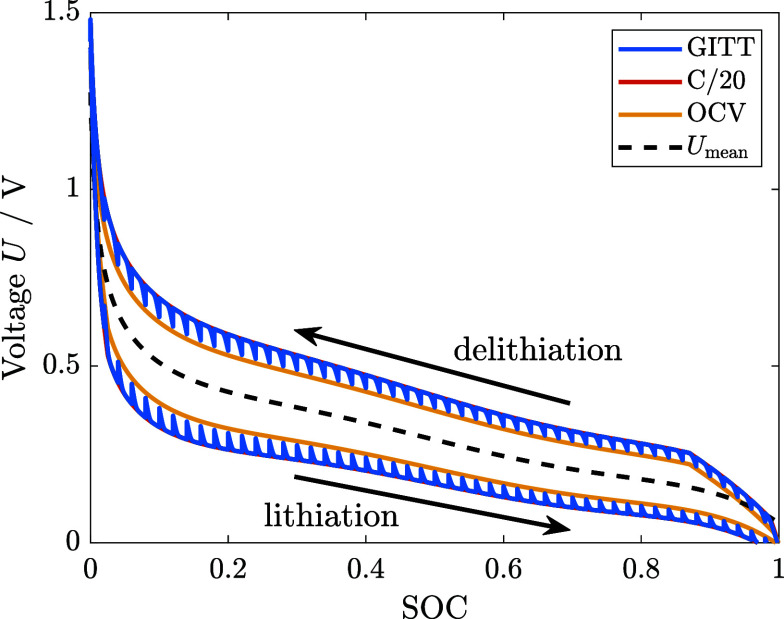
Voltages according to the presented reduced model during GITT, C/20 cycling, and after 12 h relaxation periods. The dashed black line depicts the mean experimental OCV measured for a silicon anode in ref.^8^.

In Figure 2, we depict the voltage profile predicted by the reduced model for a GITT procedure with (de)lithiation steps of  with C/20 and relaxation periods of 3 h. Furthermore, the figure shows the voltage during C/20 cycling and after 12 h relaxation periods. The dashed black line depicts the fitted mean experimental OCV curve *U*_mean_ between the measured lithiation and delithiation voltage after 3 h rest period for a pure silicon anode from ref.^8^ used as true OCV curve for the simulations. Note that the mean experimental OCV does not coincide with the mean value of the simulated OCV curves in the extreme SOC regimes due to an asymmetric stress situation discussed in detail in Section 4.3.

## 3 Computational and Experimental Details

### 3.1 Simulation Setup

Our simulations describe the behavior of a silicon nanoparticle anode with a single-particle model. We implement our model in MATLAB using a finite-difference approach by discretizing the radial dimension. To solve the set of differential eqs 6–8, 11 and 12, we use the solver ode15i. The variables inside the silicon core are the concentration of lithium , the deformed radius *r*_core_, and the radial component of the plastic deformation  of each silicon core element. The variables inside the shell are the deformed radius *r*_shell_ and the radial component of the plastic deformation  of each shell element.

### 3.2 Material Parameters

We adopt the parameters from our previous publication^21^ and adapt where necessary. Particularly, we consider a stiff, inorganic (SEI) shell with Young’s modulus of  compatible with experiments.^45,46^ The viscosity of the (inner SEI) shell is considered as a fit value and may range from  for a highly viscous polymer^47^ to  for silicon oxide.^48−50^

### 3.3 Experimental Setup

The experiments analyzed in this study have been performed and published by Wycisk et al.^11^ at Mercedes following discussions with the authors of this manuscript. The publication discusses full-cell voltage measurements with an NMC811 cathode and anodes with varying contents of silicon active material. Here, we constrain solely to the experimental results discussing anodes with pure silicon active material. The silicon anode consists of silicon nanoparticles attached to a conductive carbon network discussed as “silicon–carbon composite granules” in ref.^51^. We summarize the experimental and our simulation protocols in Section SIII but refer to the experimental publication for the experimental details.^11^

Throughout this manuscript, we consider voltages from the anode perspective and calculate voltage differences to the mean OCV, . For comparison, the voltage difference for the performed full-cell measurements is calculated as .

## 4 Results and Discussion

### 4.1 Experimental Results: Logarithmic Voltage Relaxation

**Figure 3:**
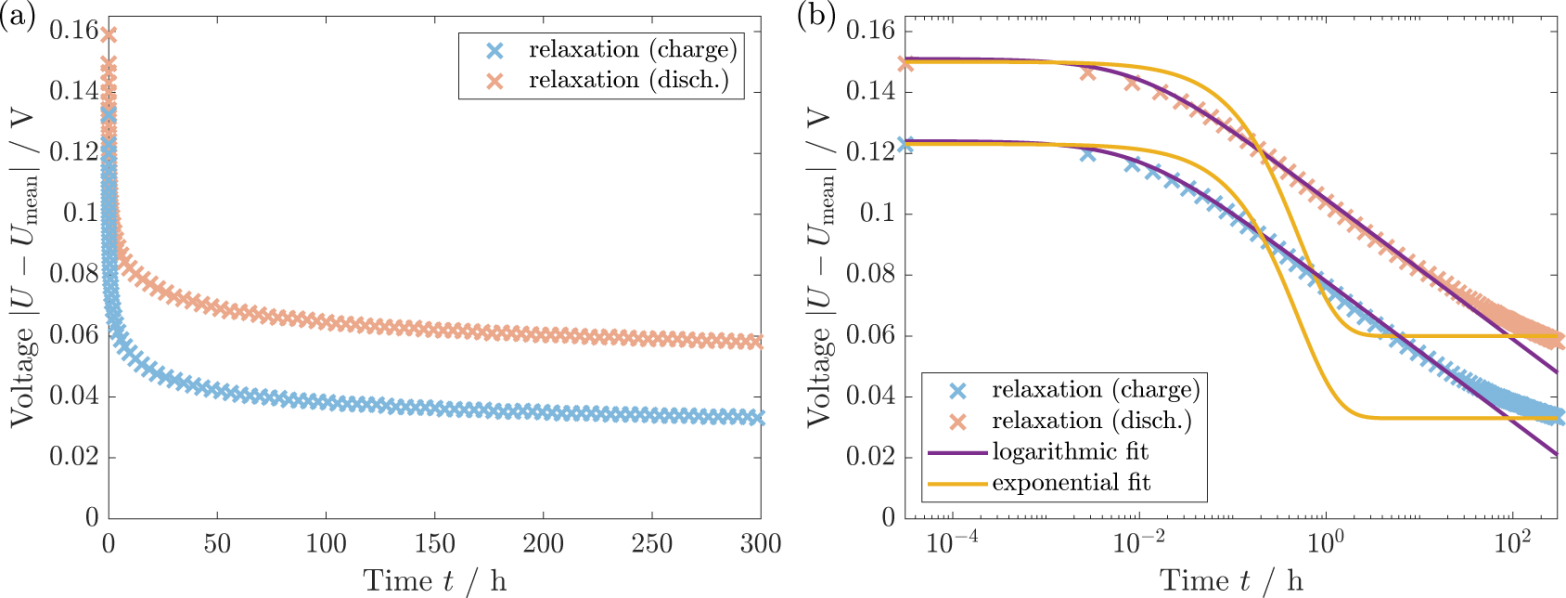
(a) Experimental voltage relaxation of silicon at SOC = 0.3 over 300 h after a charge and discharge period (protocol SIII A).^11^ (b) The semilogarithmic plot unveils the logarithmic voltage relaxation behavior.

First, we analyze the long-time relaxation experiment performed by Wycisk et al.^11^ following the protocol described in Section SIII A. In Figure 3, we depict the voltage relaxation at the same SOC measured once in charge and once in discharge direction.

Interestingly, the authors of ref.^11^ find that even after 300 h of rest, the voltage depicted in Figure 3a is not completely relaxed. Therefore, the true OCV value deviates from the relaxed voltage after 300 h and strongly deviates from standard GITT measurements with only a few hours of voltage relaxation. The authors of ref.^11^ exclude degradation or self-discharge due to the similar voltage relaxation profiles after the charge and discharge period. However, the mean value of the relaxed voltage after 300 h varies from the mean OCV measured with GITT for C/20 and 12 h rest periods. Therefore, the relaxed voltages after lithiation and delithiation reveal different values with a deviation of 0.03 V. The difference can occur on the one hand due to cell-to-cell deviations of the experimental cells. On the other hand, a minor drift in the absolute SOC estimation of only 2% is already sufficient to create such a small voltage difference.

Here, we investigate the voltage relaxation profile in detail again. In Figure 3b, we show the voltage relaxation over time as a semilogarithmic plot. Apparently, the voltage relaxation profile does not follow a typical exponential relaxation behavior, as illustrated in yellow. We identify a linear regime in the semilogarithmic plot and fit a logarithmic function to the experimental data. The logarithmic fit agrees with the experimental data in a wide range of times *t* < 20 h. Only for times larger than 20 h, the voltage relaxation slightly diminishes, leaving the logarithmic regime. This is expected as logarithmic behavior would diverge for large times. The logarithmic voltage relaxation found in the experiment agrees with the experimentally observed voltage relaxation of silicon thin-film electrodes in ref.^20^.

Regarding the hysteresis hypotheses in literature, the experimentally identified logarithmic voltage relaxation is in stark contrast to diffusional effects. Overpotentials due to diffusion would reveal an exponential voltage relaxation behavior, which cannot reproduce the experimental data as illustrated in Figure 3b. Moreover, reaction kinetics as the reason for the voltage hysteresis and relaxation would require unreasonable parameter values of the exchange current density and the anodic and cathodic transfer coefficients .^20^ Therefore, the observed logarithmic voltage relaxation provides clear support for the mechanical origin of the silicon voltage hysteresis.

### 4.2 Simulation Results: Slow Voltage Relaxation

As discussed in Section 2.1, the silicon OCV hysteresis results from elastoplastic stress generated by the shell, and the enlarged voltage hysteresis during cycling results from viscous shell stress acting on the particle core.^21^ A simple Newtonian viscosity model, , with constant viscosity η_shell_ would imply exponential voltage relaxation behavior during rest contrasting the experimental observations. Due to the large stresses inside the shell, the Newtonian model is not suitable for describing the viscous behavior. Instead, for large stresses, the strain rate is known to depend exponentially on the stress, leading to a logarithmic stress relaxation behavior. Therefore, we use the established Garofalo law given in eq 10 to describe both regimes.

**Figure 4:**
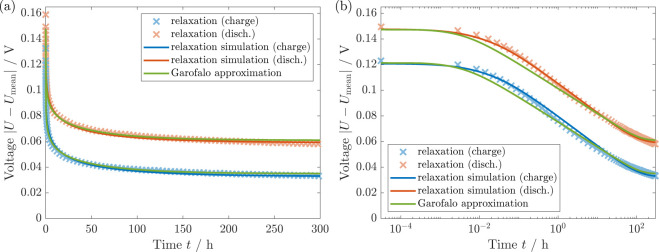
(a) Voltage relaxation of silicon at SOC = 0.3 over 300 h (protocol SIII A). Comparison of simulation, experiment,^11^ and the analytical Garofalo approximation. (b) The semilogarithmic plot shows agreement of the various curves.

Using the Garofalo model, Figure 4 depicts our simulation results in comparison to the experimental data. The parameters are given in Table S1. We shift our simulations to match the observed voltage after relaxation. The simulations reproduce the voltage relaxation profiles after the charge and discharge period. In particular, the simulation using the Garofalo law describes both the logarithmic relaxation regime as well as the decreasing relaxation after 20 h. The agreement confirms the explanation of the silicon voltage hysteresis by a visco-elastoplastic shell behavior.

To validate our simulation results, Figure 4 compares our simulation and the experiment to the analytical approximation presented in Section 2.2. The analytical approximation for the voltage relaxation with Garofalo law viscosity reveals a similar logarithmic relaxation regime followed by a slowed relaxation. Thus, the specific trends observed for our simulation and the analytical approximation agree while the actual values deviate slightly. Nevertheless, as the analytical approach relies on several assumptions and approximations, the similarity of the voltage profile supports our simulation results.

### 4.3 OCV and Cycling Voltage Hysteresis

**Figure 5:**
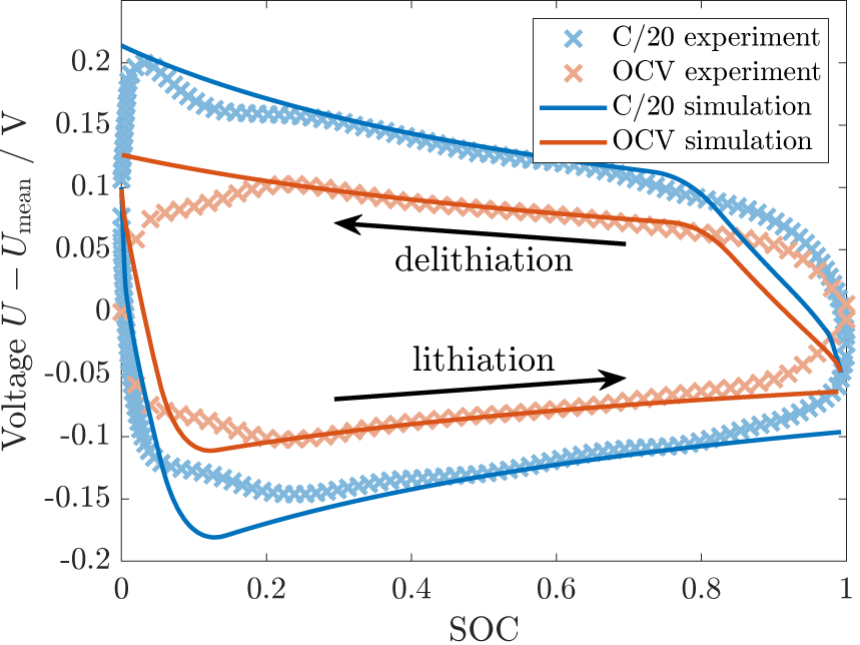
C/20 and open-circuit voltage hysteresis after 12 h relaxation in simulation and experiment (protocol SIII B).^11^

Silicon anodes are generally known to show a significant voltage hysteresis. In Figure 5, we depict the experimental OCV hysteresis after relaxation and the enlarged voltage hysteresis during slow cycling.^11^ We describe the protocol in Section SIII B. To check the consistency of our model with the experimental voltage hysteresis, Figure 5 shows the simulation of the anode voltage during slow cycling and the OCV after relaxation depending on the SOC for the parameters obtained from the voltage relaxation behavior. The illustrated voltages describe the influence on the silicon anode voltage. Hence, the voltage decreases during lithiation due to compressive stress and increases during delithiation due to tensile stress. The simulation results in Figure 5 reveal a significant OCV hysteresis resulting from the elastoplastic contribution. Furthermore, the simulation shows an enlarged hysteresis during cycling caused by viscous stress.

The comparison of the cycling and relaxed voltages reveals a good agreement between simulation and experiment in a wide SOC regime. However, our simulation and the experiment deviate slightly at both extremes, SOC < 0.2 and SOC > 0.8. This disagreement results at least partially from the determination of the true, stress-free OCV curve as the mean between lithiation and delithiation OCV. At very high SOC, the elastoplastically generated compressive stress during lithiation is fully developed, while the tensile stress during the following delithiation has to build up gradually after the change of direction. Analogously, the tensile stress during delithiation is fully developed, while the compressive stress during the following lithiation has to build up gradually after the change of direction at low SOC. Therefore, the mean value between the lithiation and delithiation OCV at both extremes is not stress-free. Its consideration as true, stress-free OCV in the simulation leads to an apparent deviation. In the Supporting Information, we discuss a corrected OCV curve assuming a constant hysteresis size in the extreme SOC regimes. Figure S3 reveals a better agreement between simulation and experiment in the extreme SOC regimes compared to Figure 5. Note that the stress asymmetry in the extreme SOC regimes is generated by the elastoplastic behavior of the shell responsible for the OCV hysteresis. The asymmetry does not result from the viscous behavior, causing the enlarged hysteresis during slow cycling and the voltage relaxation.

In our previous publication,^21^ we compared our simulation to the GITT measurement performed for a silicon half cell by Pan et al.^8,9^ The cells differ significantly from the cells investigated by Wycisk et al.^11^ due to a presumably different silicon raw material and electrolyte composition. Nevertheless, we compare our new model and the parameters obtained from the voltage relaxation^11^ to the GITT measurement^8,9^ in Section SV. Figure S4 shows a reasonable match of simulation and experiment considering the full GITT procedure as well as a single GITT pulse. The agreement confirms the applicability of our chemo-mechanical model to GITT measurements with different cells.

### 4.4 C-Rate Dependence of Voltage Hysteresis

**Figure 6:**
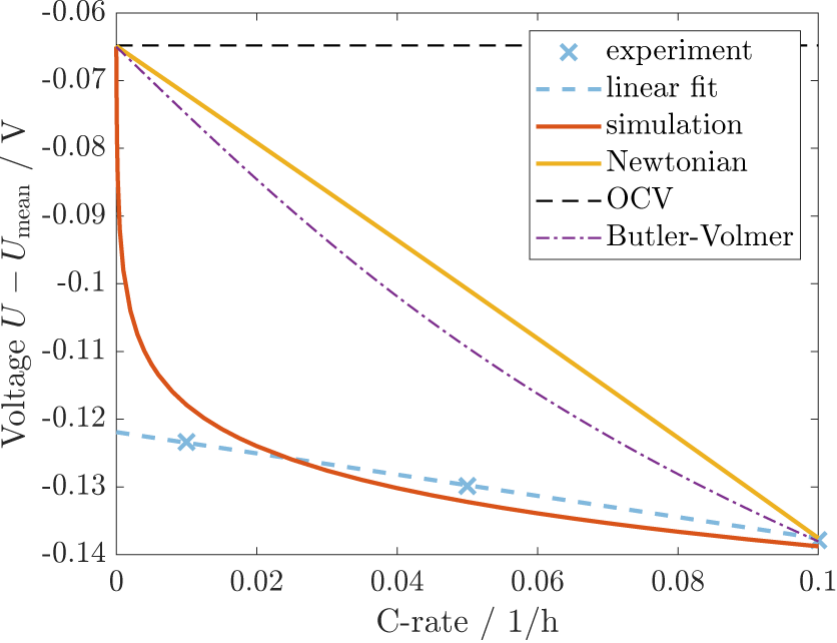
C-rate dependence of voltage hysteresis at SOC = 0.5 in simulation and experiment (protocol SIII C).^11^

The experimental data obtained by Wycisk et al.^11^ also cover the C-rate dependence of the voltage difference between the cycling voltage and the relaxed voltage after 12 h at SOC = 0.5 following the protocol given in Section SIII C. As displayed in Figure 6, the data reveal a linear dependence of the voltage on the C-rate. However, extrapolating this linear dependence to zero current results in a significant voltage offset compared to the OCV after infinite relaxation time. This offset would imply an enlarged hysteresis even for infinitely slow cycling, which is unexpected. Therefore, the authors conclude that the voltage will depart from the linear trend at particularly low C-rates.

The Newtonian viscosity model has a linear relation between the strain rate and the viscous stress. Hence, the size of the additional voltage hysteresis is linearly dependent on the C-rate as illustrated in yellow in Figure 6. However, the Newtonian model explains no voltage offset, and the slope disagrees with the experiment when matching the hysteresis size at C/10.

In comparison to the experimental and the Newtonian C-rate dependence, Figure 6 also depicts the simulated C-rate dependence. The inverse hyperbolic sine in eq 10 determines the C-rate dependence of the viscous stress and, consequently, the C-rate dependence of the additional voltage hysteresis during cycling. Thus, the simulation reveals a nonlinear dependence of the voltage on the current. Nonetheless, after a swift increase of the voltage at current rates smaller C/100, the increase slows down, approaching an almost linear trend with small curvature. Although the three experimental data points follow the linear trend exactly, we assume that our simulation is in reasonable agreement with the experiment and additionally describes the transition to vanishing voltage at zero current. We expect that more experimental data points particularly at low C-rates might indicate a curvature and deviation from the linear trend.

Concerning diffusion and reaction overpotentials as alternative hysteresis hypotheses stated in literature, diffusion overpotentials inhere a linear dependence on the C-rate without offset, coinciding with the curve for Newtonian viscosity (yellow) in Figure 6. Further, reaction overpotentials expressed by the Butler–Volmer equation with typical symmetry factor α = 0.5 show only a slight curvature (purple) in Figure 6. Considering a parameter variation, Figure S5 demonstrates that unreasonable anodic transfer coefficients  are necessary to approach the experimentally observed C-rate dependence. Therefore, neither diffusion nor reaction overpotentials can reasonably reproduce the experimentally observed dependence on the C-rate. This demonstrates once again the insufficiency of transport and reaction overpotentials for explaining the silicon voltage hysteresis, thereby promoting our chemo-mechanical explanation.

### 4.5 Voltage Transition Profiles

Another interesting behavior is the silicon anode voltage profile of transitions between cycling and rest periods. In the following, we discuss the features of different transitions and compare our simulation to the experimental data from ref.^11^ wherever possible.

**Figure 7:**
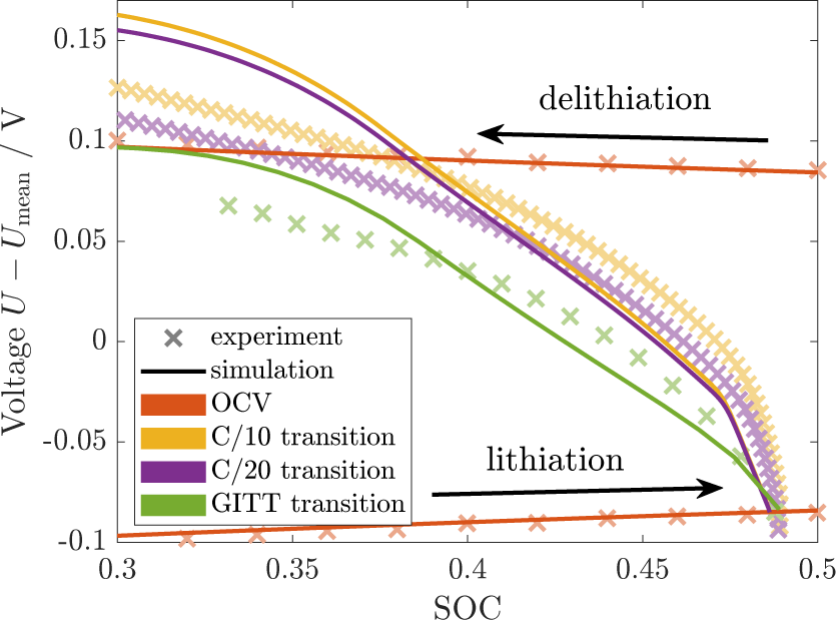
Voltage transition from lithiation to delithiation in simulation and experiment (protocol SIII D).^11^

First, we investigate the transition profile between lithiation and delithiation according to protocol SIII D. In Figure 7, we show the delithiation with either C/10, C/20, or GITT procedure after a continuous lithiation and rest period. For reference, the figure also includes the simulated and measured lithiation and delithiation OCV curves from Figure 5, which almost coincide in the depicted regime . All experimental data^11^ reveal a smooth transition between the lithiation and delithiation voltage. The slope of the voltage profiles is large directly after the change of direction and slows down gradually when approaching the delithiation voltage.

The numerical results are depicted in Figure 7 compared to the experiment. When switching the current direction from lithiation to delithiation, the simulated voltage profiles for C/10 (yellow) and C/20 (purple) currents reveal three regimes. Immediately after the change of direction, the voltage shows a steep increase for a small span of  attributed to the rapid buildup of viscous stress. Afterward, for a range of , a constant, moderate voltage slope demonstrates the decrease of compressive elastic stress and the subsequent increase in tensile elastic stress. In the third regime, the slope slows down, and the voltage approaches a maximum value when reaching the yield criterion for plasticity. The higher current C/10 shows a slightly faster voltage transition compared to the lower current C/20. For the GITT transition curve (green), the relaxation of viscous stress during the rest periods suppresses the viscous regime after the change of direction. Contrary to the simulation, the experimental curves do not reveal clearly defined regimes but are in line with the general trend of a rapid voltage increase after the change of direction followed by an attenuated transition to the delithiation voltage curve. The much smoother experimental results compared to our simulation are expected as we consider only a single-particle model but the detailed features average out in the multi-particle experiment. Thus, we conclude that our simulation result agrees reasonably with the experimental measurement.

In Section SVII, we evaluate the behavior of an interrupted lithiation pulse for different C-rates and at different SOC values. All voltage profiles in Figures S6 and S7 show a steep slope at the beginning of the pulses, revealing the increase in viscous stress followed by a slower convergence to the lithiation voltage, indicating elastoplastic behavior. The similar voltage profiles for different C-rates indicate that the voltage transition needs a certain amount of charge throughput or SOC change Δ*S*OC in accordance with the experimental results from ref.^11^ for a blended graphite-silicon anode. Additionally, the voltage profiles at different SOC values in Figure S6 show that the general trends of the chemo-mechanical simulation agree with the ones of the experiment. However, all experimental curves show an overshoot instead of a smooth convergence to the lithiation voltage, which is not visible in our simulations. In terms of mechanics, this overshoot might result from a thixotropic behavior of the shell as discussed in the Supporting Information.

Another voltage hysteresis effect measured for silicon anodes is a pronounced relaxation during rest observed for higher applied currents.^11,52^ Higher C-rates show an increased voltage hysteresis during cycling in agreement with our viscosity model. However, this dependence surprisingly inverts after relaxation. This phenomenon is not captured in our chemo-mechanical single-particle model. Therefore, we support the interpretation as a multi-particle effect^11^ and add a mechanical explanation. For fast charging, the silicon particles inside the anode will lithiate more inhomogeneously, causing enhanced plastic flow of the shell around particles with a higher lithiation level. During the subsequent rest period, the silicon particles with initially higher lithiation degrees delithiate slightly. The shrinkage of those particles reduces the remaining compressive stress, while the stress in the particles with initially lower lithiation levels can not exceed the yield stress for plastic flow. Hence, this multi-particle effect can reduce the mean stress hysteresis inside the silicon anode and, consequently, the voltage hysteresis after relaxation.

**Figure 8:**
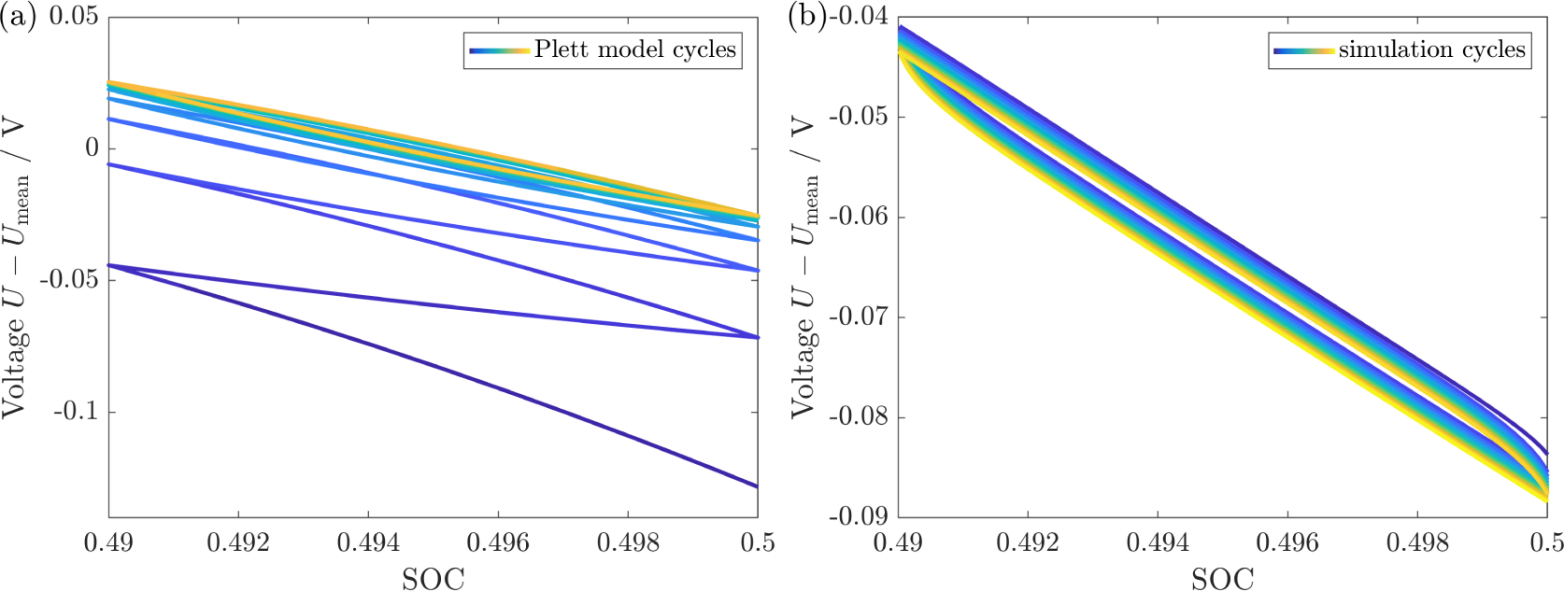
Voltage for alternating lithiation and delithiation pulses with  (protocol SIII F) for (a) the phenomenological Plett model and (b) our chemo-mechanical simulation.

Finally, we estimate the voltage transition behavior for alternating short lithiation and delithiation pulses following protocol SIII F. The silicon voltage hysteresis is often described empirically with the Plett model presented in Section SVIII.^53−55^ In Figure 8a, we depict the behavior for alternating pulses with Δ*S*OC = 0.01 predicted by the empirical Plett model with the parameters adjusted to fit the experimental voltage hysteresis. The Plett model does not reveal a constant hysteresis behavior during 10 subsequent cycles but rather approaches the mean OCV within the first cycles and then describes a hysteresis around it. Additionally, the Plett model is not able to account for a relaxation phase without a change in SOC. In contrast, Figure 8b shows the simulation of alternating pulses, which reveal a permanent hysteresis during 10 subsequent cycles. Only the very first pulse initially shows a slightly different behavior with an enlarged hysteresis size because of a different stress state in the initial situation after the 12 h relaxation period. We know that experiments show a permanent hysteresis behavior upon alternate lithiation and delithiation pulses in line with our simulation result. Thus, we conclude that our chemo-mechanical core–shell model outperforms the empirical Plett model in the description of voltage hysteresis phenomena.

## 5 Conclusions

Detailed analysis of the silicon voltage hysteresis experiments performed by Wycisk et al.^11^ reveals a slow, non-exponential voltage relaxation. We identify a logarithmic voltage relaxation for a wide range of times and a transition to exponential relaxation for larger times due to the divergence of the logarithmic behavior. With a chemo-mechanical core–shell model, we have illustrated that the visco-elastoplastic shell behavior following the Garofalo law or inverse hyperbolic sine law for viscosity can accurately describe the voltage relaxation of a silicon anode over the whole time span. Our simulations also reproduce the observed voltage hysteresis and GITT measurement with the parameters obtained from the relaxation experiment. Our core–shell model can be interpreted as silicon nanoparticles covered by SEI but can also portray active silicon nanodomains within larger silicon particles.

Additionally, the Garofalo viscosity model can approach the experimentally observed C-rate dependence of the cycling voltage hysteresis. The inverse hyperbolic sine behaves approximately linear in a wide span of C-rates but shows a kink and reveals vanishing additional voltage hysteresis at zero current. Therefore, the Garofalo law viscosity model fits much better to the C-rate dependence than Newtonian viscosity, which reveals a proportional relation between the voltage and the applied C-rate.

With a focus on the voltage transition behavior between lithiation and delithiation, the presented chemo-mechanical model can adequately describe the general trends of an initially fast voltage transition followed by an attenuated convergence to the delithiation voltage curve. The interplay of viscous, elastic, and plastic contributions to the simulated voltage explains this voltage profile. Furthermore, our model reasonably describes the lithiation behavior after a rest period. Thus, our chemo-mechanical core–shell model outperforms the empirical Plett model regarding physical understanding as well as the description of the various features of the hysteresis phenomenon.

The overall accordance of our simulations to experimental results supports our chemo-mechanical explanation of the voltage hysteresis presented initially in ref.^21^. The description of the viscous behavior using the Garofalo law is more suitable than linear Newtonian viscosity because of the large stresses reached inside the shell. In conclusion, we have demonstrated that our physical model presents a consistent picture of the various features of the silicon voltage hysteresis phenomenon.

## Supporting Information Available

Analytical approximation for the voltage relaxation in the extremestress regimes; derivation of reduced model; detailed experimental and simulation protocols; corrected open-circuit voltage (OCV) curve; comparison with GITT measurement; C-rate dependence of reaction overpotentials; voltage profiles of lithiation pulses; Plett model; parameters (PDF)
