# Supplementary material for: Slow Voltage Relaxation of Silicon Nanoparticles with a Chemo-Mechanical Core–Shell Model
Source: ACS Appl Mater Interfaces. 2024 Nov 26;16(49):67609–19. doi: 10.1021/acsami.4c12976 (PMC11647879; doi:10.1021/acsami.4c12976)
Supplement: Supplementary file 1 — am4c12976_si_001.pdf [file am4c12976_si_001.pdf]

Supporting Information: Slow Voltage Relaxation of Silicon Nanoparticles with a Chemo-Mechanical Core-Shell Model

Lukas Köbbing, Yannick Kuhn, and Birger Horstmann*
Institute of Engineering Thermodynamics, German Aerospace Center (DLR), Wilhelm-Runge-Straße 10, 89081 Ulm, Germany
Helmholtz Institute Ulm (HIU), Helmholtzstraße 11, 89081 Ulm, Germany and
Faculty of Natural Sciences, Ulm University, Albert-Einstein-Allee 47, 89081 Ulm, Germany

SI. ANALYTICAL APPROXIMATION FOR THE VOLTAGE RELAXATION IN THE EXTREME STRESS REGIMES

The time evolution of the general stress relaxation behavior during rest is derived in the manuscript as

$$\frac{\mathrm{d}\sigma_{\mathrm{ev}}}{\mathrm{d}t} = -E_{\mathrm{core}}\frac{1}{\tau\lambda_{\mathrm{ch}}^2}\sinh\left(\frac{\alpha\lambda_{\mathrm{ch}}^3\sigma_{\mathrm{ev}}}{\sigma_{\mathrm{ref}}}\right) \tag{S1}$$

with the analytical solution

$$\sigma_{\mathrm{ev}} = \frac{2\sigma_{\mathrm{ref}}}{\alpha\lambda_{\mathrm{ch}}^3}\cdot\operatorname{atanh}\left(C\cdot\exp\left(-\frac{E_{\mathrm{core}}\alpha\lambda_{\mathrm{ch}}}{\tau\sigma_{\mathrm{ref}}}t\right)\right). \tag{S2}$$

To understand the origin and the regimes of the convoluted functional behavior in Equation (S2), we analyze the relaxation behavior in the limits of low and large stress magnitudes in the following.

First, to solve the differential equation (S1) analytically in the limit of large compressive stress, i.e.

$$\frac{\sigma_{\mathrm{ev}}}{\sigma_{\mathrm{ref}}} \ll -1, \tag{S3}$$

the hyperbolic sine can be approximated by

$$\sinh x = \frac{1}{2}\left(\mathrm{e}^{x} - \mathrm{e}^{-x}\right) \approx -\frac{1}{2}\mathrm{e}^{-x}. \tag{S4}$$

Thus, the differential equation in the large compressive stress regime reads

$$\frac{\mathrm{d}\sigma_{\mathrm{ev}}}{\mathrm{d}t} = -\frac{E_{\mathrm{core}}}{\tau\lambda_{\mathrm{ch}}^2}\cdot\left(-\frac{1}{2}\right)\exp\left(-\frac{\alpha\lambda_{\mathrm{ch}}^3\sigma_{\mathrm{ev}}}{\sigma_{\mathrm{ref}}}\right). \tag{S5}$$

The analytical solution for this differential equation is

$$\sigma_{\mathrm{ev}} = \frac{\sigma_{\mathrm{ref}}}{\alpha\lambda_{\mathrm{ch}}^3}\cdot\ln\left(\frac{E_{\mathrm{core}}\alpha\lambda_{\mathrm{ch}}}{2\tau\sigma_{\mathrm{ref}}}t + C_{\mathrm{exp}}\right). \tag{S6}$$

Using the chemo-mechanical coupling $\Delta U = v_{\mathrm{Li}}\sigma/F$, the voltage relaxation reads

$$\Delta U_{\mathrm{ev}} = \frac{v_{\mathrm{Li}}\sigma_{\mathrm{ref}}}{\alpha F\lambda_{\mathrm{ch}}^3}\cdot\ln\left(\frac{E_{\mathrm{core}}\alpha\lambda_{\mathrm{ch}}}{2\tau\sigma_{\mathrm{ref}}}t + C_{\mathrm{exp}}\right). \tag{S7}$$

The integration constant C_{exp} has to be determined from the boundary condition as

$$C_{\mathrm{exp}} = \exp\left(\frac{\alpha\lambda_{\mathrm{ch}}^3\sigma_0}{\sigma_{\mathrm{ref}}}\right). \tag{S8}$$

* birger.horstmann@dlr.de

Second, to solve the differential equation analytically in the low compressive stress limit with

$$-1 < \frac{\sigma_{\mathrm{ev}}}{\sigma_{\mathrm{ref}}} \leq 0 \tag{S9}$$

we approximate the hyperbolic sine as

$$\sinh x = \frac{1}{2}\left(\mathrm{e}^{x} - \mathrm{e}^{-x}\right) \approx x. \tag{S10}$$

Therefore, the differential equation (S1) in the low compressive stress limit reads

$$\frac{\mathrm{d}\sigma_{\mathrm{ev}}}{\mathrm{d}t} = -\frac{E_{\mathrm{core}}}{\tau} \cdot \frac{\alpha\lambda_{\mathrm{ch}}\sigma_{\mathrm{ev}}}{\sigma_{\mathrm{ref}}}. \tag{S11}$$

This equation describes Newtonian viscosity with $\eta = \tau\sigma_{\mathrm{ref}}$. The analytical solution states

$$\sigma_{\mathrm{ev}} = C_{\mathrm{lin}} \cdot \exp\left(-\frac{E_{\mathrm{core}}\alpha\lambda_{\mathrm{ch}}}{\tau\sigma_{\mathrm{ref}}}t\right). \tag{S12}$$

The voltage relaxation reads

$$\Delta U_{\mathrm{ev}} = \frac{v_{\mathrm{Li}}C_{\mathrm{lin}}}{F} \cdot \exp\left(-\frac{E_{\mathrm{core}}\alpha\lambda_{\mathrm{ch}}}{\tau\sigma_{\mathrm{ref}}}t\right). \tag{S13}$$

This time, the constant C_{lin} has to be determined from the boundary condition at the time entering the low compressive stress limit.

Thus, the general solution describes logarithmic behavior in the large stress limit but exponential relaxation behavior in the low stress limit. We depict the approximations for the large and low stress limits in Figure S1 in comparison to the general approximation and the experimental data from Ref. [1].

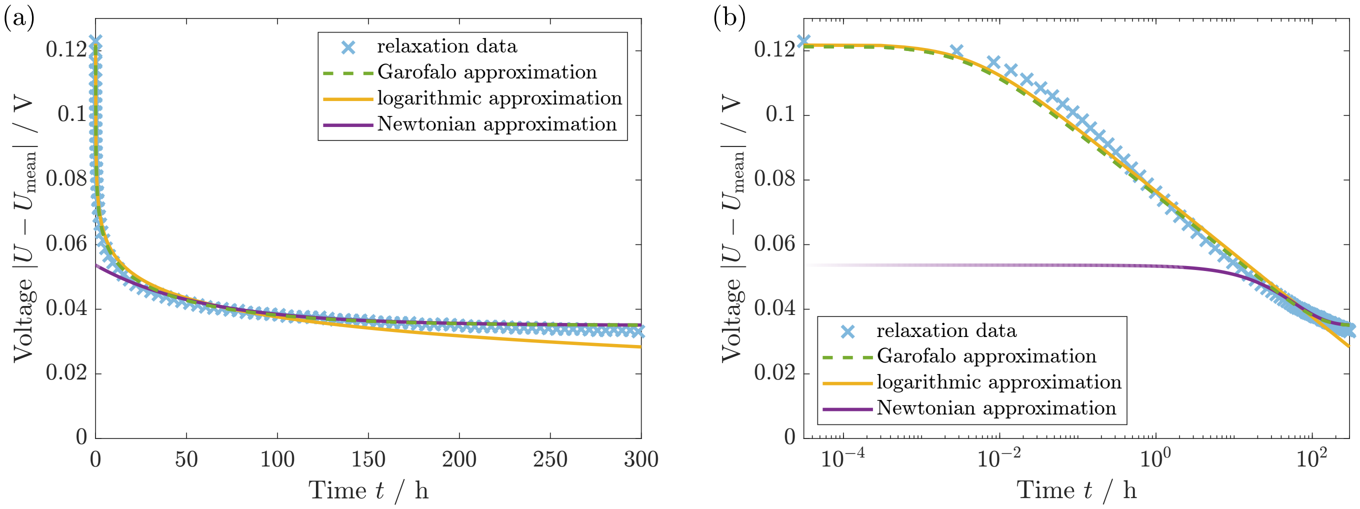

Figure S1. (a) Voltage relaxation of silicon described by the analytical approximations in comparison to the experiment [1]. (b) Semi-logarithmic plot of approximations and experiment [1].

SII. DERIVATION OF REDUCED MODEL

First, we assume volume conservation of the shell

$$1 = J_{\mathrm{shell}} = \mathbf{F}_{\mathrm{shell,rr}} \mathbf{F}^2_{\mathrm{shell},\varphi\varphi}. \tag{S14}$$

The tangential deformation of the shell is determined by the total expansion of the particle dominated by the chemical deformation

$$\mathbf{F}_{\mathrm{shell,rr}} = \mathbf{F}^{-2}_{\mathrm{shell},\varphi\varphi} \approx \lambda_{\mathrm{ch}}^{-2} = (1 + v_{\mathrm{Li}} c_{\mathrm{Li},0})^{-\frac{2}{3}} . \tag{S15}$$

The strain rate reads

$$\dot{\mathbf{E}}_{\mathrm{shell,rr}} = \mathbf{F}_{\mathrm{shell,rr}} \dot{\mathbf{F}}_{\mathrm{shell,rr}} = \mathbf{F}^{-2}_{\mathrm{shell},\varphi\varphi} \dot{\left(\mathbf{F}^{-2}_{\mathrm{shell},\varphi\varphi}\right)} = -2\mathbf{F}^{-5}_{\mathrm{shell},\varphi\varphi} \dot{\mathbf{F}}_{\mathrm{shell},\varphi\varphi} \approx -2\lambda_{\mathrm{ch}}^{-6} \dot{\mathbf{E}}_{\mathrm{core,rr}} \tag{S16}$$

using isotropic deformation inside the silicon particle core $\mathbf{E}_{\mathrm{core,rr}} = \mathbf{E}_{\mathrm{core},\varphi\varphi}$. In the elastic regime, the elastic stress contribution from the shell reads

$$\boldsymbol{\sigma}_{\mathrm{shell,el,rr}} = E_{\mathrm{shell}} \mathbf{E}_{\mathrm{shell,rr}}. \tag{S17}$$

The elastic stress is mainly generated during cycling by the chemical deformation rate

$$\dot{\mathbf{E}}_{\mathrm{shell,rr}} = -2\lambda_{\mathrm{ch}}^{-5} \dot{\lambda}_{\mathrm{ch}} = -\frac{2v_{\mathrm{Li}}}{3\lambda_{\mathrm{ch}}^7} \dot{c}_{\mathrm{Li},0}. \tag{S18}$$

The time evolution of the elastic stress inside the particle due to the elastic stress of the shell reads

$$\dot{\sigma}_{\mathrm{ee}} = \dot{\boldsymbol{\sigma}}_{\mathrm{shell,el,rr}} = -E_{\mathrm{shell}} \frac{2v_{\mathrm{Li}}}{3\lambda_{\mathrm{ch}}^7} \dot{c}_{\mathrm{Li},0}. \tag{S19}$$

The yield criterion is given by the deviatoric Mandel stress. Due to the constant shell volume, Mandel stress equals Cauchy stress

$$\mathbf{M}^{\mathrm{dev}}_{\mathrm{shell,el}} = \mathbf{M}_{\mathrm{shell,el}} - \frac{1}{3} \operatorname{tr} \mathbf{M}_{\mathrm{shell,el}} = \boldsymbol{\sigma}_{\mathrm{shell,el}} - \frac{1}{3} \operatorname{tr} \boldsymbol{\sigma}_{\mathrm{shell,el}} \tag{S20}$$

and

$$\left|\mathbf{M}^{\mathrm{dev}}_{\mathrm{shell,el}}\right|^2 = \left(\frac{2}{3}\left(\boldsymbol{\sigma}_{\mathrm{shell,el,rr}} - \boldsymbol{\sigma}_{\mathrm{shell,el},\varphi\varphi}\right)\right)^2 + 2\left(-\frac{1}{3}\left(\boldsymbol{\sigma}_{\mathrm{shell,el,rr}} - \boldsymbol{\sigma}_{\mathrm{shell,el},\varphi\varphi}\right)\right)^2 = \frac{2}{3}\left(\boldsymbol{\sigma}_{\mathrm{shell,el,rr}} - \boldsymbol{\sigma}_{\mathrm{shell,el},\varphi\varphi}\right)^2 . \tag{S21}$$

The empirical relation from our simulations $\mathbf{P}_{\mathrm{shell,rr}} \approx -\frac{2L_{\mathrm{shell}}/R_{\mathrm{core}}}{1-L_{\mathrm{shell}}/R_{\mathrm{core}}} \mathbf{P}_{\mathrm{shell},\varphi\varphi}$ implies $\boldsymbol{\sigma}_{\mathrm{shell},\varphi\varphi} = -\frac{1}{2}(R_{\mathrm{core}}/L_{\mathrm{shell}} - 1)\lambda_{\mathrm{ch}}^3 \boldsymbol{\sigma}_{\mathrm{shell,rr}}$ and the yield condition

$$f = \frac{\frac{3}{2}\left|\mathbf{M}^{\mathrm{dev}}_{\mathrm{shell,el}}\right|^2}{\sigma^2_{\mathrm{Y,shell}}} - 1 = \frac{\left(\boldsymbol{\sigma}_{\mathrm{shell,el,rr}}(1 + \frac{1}{2}(R_{\mathrm{core}}/L_{\mathrm{shell}} - 1)\lambda_{\mathrm{ch}}^3)\right)^2}{\sigma^2_{\mathrm{Y,shell}}} - 1 \leq 0. \tag{S22}$$

When the yield criterion is reached, plastic flow determines the deformation and the stress follows the yield surface

$$\dot{\sigma}_{\mathrm{ee}} = \dot{\boldsymbol{\sigma}}_{\mathrm{shell,el,rr}} = \sigma_{\mathrm{Y,shell}} \left(1 + \frac{1}{2}\left(\frac{R_{\mathrm{core}}}{L_{\mathrm{shell}}} - 1\right)\lambda_{\mathrm{ch}}^3\right)^{-2} \frac{1}{2}\left(\frac{R_{\mathrm{core}}}{L_{\mathrm{shell}}} - 1\right) v_{\mathrm{Li}} \left|\dot{c}_{\mathrm{Li},0}\right| . \tag{S23}$$

For the viscous part of the stress, we take the time evolution of the elastic silicon core stress due to viscous stress of the shell

$$\dot{\sigma}_{\mathrm{ev}} = E_{\mathrm{core}} \dot{\mathbf{E}}_{\mathrm{core,ev,rr}}. \tag{S24}$$

We describe the deformation of the silicon particle with a multiplicative approach

$$\mathbf{F}_{\mathrm{core}} = \mathbf{F}_{\mathrm{core,el}} \mathbf{F}_{\mathrm{core,ch}} = \mathbf{F}_{\mathrm{core,ee}} \mathbf{F}_{\mathrm{core,ev}} \mathbf{F}_{\mathrm{core,ch}} \approx \mathbf{F}_{\mathrm{core,ev}} \mathbf{F}_{\mathrm{core,ch}}. \tag{S25}$$

Therefore, the elastic strain rate due to viscosity reads

$$\dot{\mathbf{E}}_{\text{core,ev}} = \mathbf{F}_{\text{core,ev}} \dot{\mathbf{F}}_{\text{core,ev}} \approx \dot{\mathbf{F}}_{\text{core,ev}} \approx \frac{\dot{\mathbf{F}}_{\text{core}}}{\lambda_{\text{ch}}} - \frac{\dot{\mathbf{F}}_{\text{core,ch}}}{\lambda_{\text{ch}}}. \tag{S26}$$

The viscous stress contribution is determined by the Garofalo law

$$\boldsymbol{\sigma}_{\text{shell,visc}} = \sigma_{\text{ref}} \operatorname{asinh}\left(\tau \dot{\mathbf{E}}_{\text{shell}}\right). \tag{S27}$$

Thus, the stress evolution reads

$$\dot{\sigma}_{\text{ev}} = E_{\text{core}} \dot{\mathbf{E}}_{\text{core,ev,rr}} \tag{S28}$$

$$= E_{\text{core}} \left(\frac{\dot{\mathbf{F}}_{\text{core,rr}}}{\lambda_{\text{ch}}} - \frac{\dot{\mathbf{F}}_{\text{core,ch}}}{\lambda_{\text{ch}}} \right) \tag{S29}$$

$$\approx E_{\text{core}} \left(\frac{\dot{\mathbf{E}}_{\text{shell},\varphi\varphi}}{\lambda_{\text{ch}}^2} - \frac{\dot{\lambda}_{\text{ch}}}{\lambda_{\text{ch}}} \right) \tag{S30}$$

$$= E_{\text{core}} \frac{1}{\tau \lambda_{\text{ch}}^2} \sinh\left(\frac{\boldsymbol{\sigma}_{\text{shell,visc},\varphi\varphi}}{\sigma_{\text{ref}}} \right) - E_{\text{core}} \frac{v_{\text{Li}}}{3\lambda_{\text{ch}}^3} \dot{c}_{\text{Li},0} \tag{S31}$$

$$= -E_{\text{core}} \frac{1}{\tau \lambda_{\text{ch}}^2} \sinh\left(\frac{\frac{1}{2}\left(\frac{R_{\text{core}}}{L_{\text{shell}}} - 1\right) \lambda_{\text{ch}}^3 \sigma_{\text{ev}}}{\sigma_{\text{ref}}} \right) - E_{\text{core}} \frac{v_{\text{Li}}}{3\lambda_{\text{ch}}^3} \dot{c}_{\text{Li},0}. \tag{S32}$$

With the chemo-mechanical coupling $\Delta U = v_{\text{Li}} \sigma / F$, the system of equations reads

$$\frac{\mathrm{d}\,\text{SOC}}{\mathrm{d}t} = \frac{\dot{c}_{\text{Li},0}}{c_{\text{Li,max}}} = \pm \frac{C_{\text{rate}}}{3600} \frac{1}{\text{s}} \tag{S33}$$

$$\frac{\mathrm{d}\,\Delta U_{\text{ee}}}{\mathrm{d}t} = \begin{cases} -E_{\text{shell}} \dfrac{2 v_{\text{Li}}^2}{3F\lambda_{\text{ch}}^7} \dot{c}_{\text{Li},0}, & \text{if } -\operatorname{sgn}\left(\dot{c}_{\text{Li},0}\right)\left(1 + \alpha \lambda_{\text{ch}}^3\right) \dfrac{F \Delta U_{\text{ee}}}{v_{\text{Li}}} < \sigma_{\text{Y,shell}} \\ \sigma_{\text{Y,shell}} \dfrac{\alpha v_{\text{Li}}^2}{F\left(1 + \alpha \lambda_{\text{ch}}^3\right)^2} \left|\dot{c}_{\text{Li},0}\right|, & \text{otherwise} \end{cases} \tag{S34}$$

$$\frac{\mathrm{d}\,\Delta U_{\text{ev}}}{\mathrm{d}t} = -E_{\text{core}} \frac{v_{\text{Li}}}{\tau F \lambda_{\text{ch}}^2} \sinh\left(\frac{\alpha \lambda_{\text{ch}}^3}{\sigma_{\text{ref}}} \frac{F \Delta U_{\text{ev}}}{v_{\text{Li}}} \right) - E_{\text{core}} \frac{v_{\text{Li}}^2}{3F\lambda_{\text{ch}}^3} \dot{c}_{\text{Li},0}. \tag{S35}$$

To simplify the equations, we define the parameter $\alpha = \frac{1}{2}\left(\frac{R_{\text{core}}}{L_{\text{shell}}} - 1\right)$.

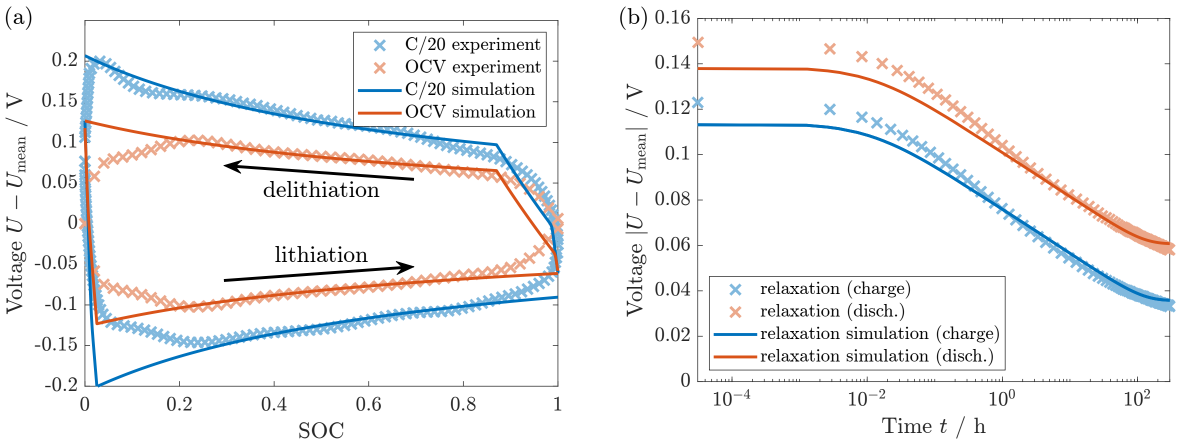

Figure S2. Results of reduced model equations in comparison to the experimental data [1]. (a) Voltage Hysteresis for C/20 current and OCV after 12 h relaxation period. (b) Voltage relaxation for 300 h after C/10 lithiation at SOC = 0.3.

SIII. DETAILED EXPERIMENTAL AND SIMULATION PROTOCOLS

In the following, we summarize the experimental and our simulation protocols but refer to the experimental publication for the experimental details [1]. We relate the measured and simulated voltages consistently to the mean value of the charging and discharging OCV obtained in Section SIII B.

A. Long-term relaxation

For the long-term relaxation experiment, the authors of Ref. [1] charge the cell with C/10 up to 30 % state-of-charge (SOC) and allow it to relax under open-circuit conditions for 300 h. Afterward, they charge the cell up to 60 % SOC, discharge to 30 % SOC, and observe the relaxation again. Thus, the authors of Ref. [1] measure the voltage relaxation at the same SOC once in charge and once in discharge direction. We apply the same protocol for our simulation of the long-term relaxation.

B. Cycling and open-circuit voltage hysteresis

Additionally, the authors of Ref. [1] measure the voltage hysteresis during slow cycling with C/20 and the OCV hysteresis between 0 % and 100 % SOC. The OCV hysteresis is obtained by the galvanostatic intermittent titration technique (GITT). The GITT protocol consists of subsequent (dis)charging steps with 2 % SOC and relaxation periods of 12 h. We simulate the cycling voltage with C/20 and the OCV after 12 h relaxation.

C. C-rate dependence of voltage hysteresis

For the C-rate dependence, the authors of Ref. [1] measure the voltage difference for varying currents during continuous charging and the relaxed voltage after 12 h at 50 % SOC. To calculate the voltage related to the mean OCV, we use the voltage after 12 h relaxation at 50 % SOC for C/20 obtained in Section SIII B. We apply the same protocol for our simulation of the C-rate dependence.

D. Voltage transition profiles

To measure the transition profile between lithiation and delithiation, the authors of Ref. [1] charge the cell up to $\mathrm{SOC} = 0.49$ with C/10 current with a following relaxation time of 12 h. Subsequently, they discharge with either C/10, C/20, or GITT steps of $\Delta\mathrm{SOC} = 0.01$ with C/10 and 12 h relaxation. We apply the same protocol for our simulation of the voltage transition.

E. Voltage profiles for interrupted lithiation

The authors of Ref. [1] measure the behavior of an interrupted lithiation pulse for different SOC values with C/20 and a rest period of 12 h. We apply the same protocol for our simulation of an interrupted simulation pulse. Furthermore, we simulate the behavior of an interrupted lithiation pulse for different C-rates after a rest period of 12 h at $\mathrm{SOC} = 0.5$.

F. Voltage profiles for alternating pulses

Finally, we simulate the voltage transition behavior for alternating pulses. We lithiate the silicon anode up to 50 % SOC with a subsequent relaxation period of 12 h. Then, we apply ten sequences of alternating delithiation and lithiation pulses with $\Delta\mathrm{SOC} = 0.01$.

SIV. CORRECTED OPEN-CIRCUIT VOLTAGE (OCV) CURVE

For simplicity and reduction of the degrees of freedom, we take the mean value of the measured OCV curves after 12 h as true OCV in our manuscript, $U_{\mathrm{mean}} = 1/2(U_{\mathrm{OCV,lithiation}} + U_{\mathrm{OCV,delithiation}})$. Nevertheless, the symmetry between lithiation and delithiation is broken at the extreme SOC values. At high SOC, elastoplastic compressive stress is fully developed during lithiation. In contrast, this compressive stress has to be reduced, and tensile stress has to build up gradually after the change in direction. At low SOC, tensile stress is fully developed during delithiation. In contrast, this tensile stress has to be reduced, and compressive stress has to build up gradually after the change in direction. To account for and estimate the significance of this effect, we define a corrected OCV curve as

$$U_{\mathrm{OCV,corr}}(\mathrm{SOC}) = \begin{cases} U_{\mathrm{OCV,delithiation}}(\mathrm{SOC}) - \left(U_{\mathrm{OCV,delithiation}}(0.2) - U_{\mathrm{mean}}(0.2)\right), & \text{if SOC} < 0.2 \\ U_{\mathrm{mean}}(\mathrm{SOC}), & \text{if } 0.2 \leq \text{SOC} \leq 0.8 \\ U_{\mathrm{OCV,lithiation}}(\mathrm{SOC}) - \left(U_{\mathrm{OCV,lithiation}}(0.8) - U_{\mathrm{mean}}(0.8)\right), & \text{if SOC} > 0.8. \end{cases} \tag{S36}$$

The corrected OCV curve equals the mean OCV curve in the regime $0.2 \leq \mathrm{SOC} \leq 0.8$. In the extreme SOC regimes, we assume that the distance between the OCV curve in the direction of fully developed stress and the true OCV stays constant. The corrected OCV curve is depicted in Figure S3(a) compared to the mean OCV curve used in our manuscript. We depict our simulation results compared to the experimental data [1] related to the corrected OCV in Figure S3(b). In comparison to Fig. 5 in our manuscript with the experiment related to the mean OCV, our simulation shows an improved agreement with the experiment related to the corrected OCV curve. Thus, a corrected OCV should be considered when a superior agreement between simulation and experiment is indispensable. Nonetheless, we use the simple approach with the mean OCV throughout our manuscript.

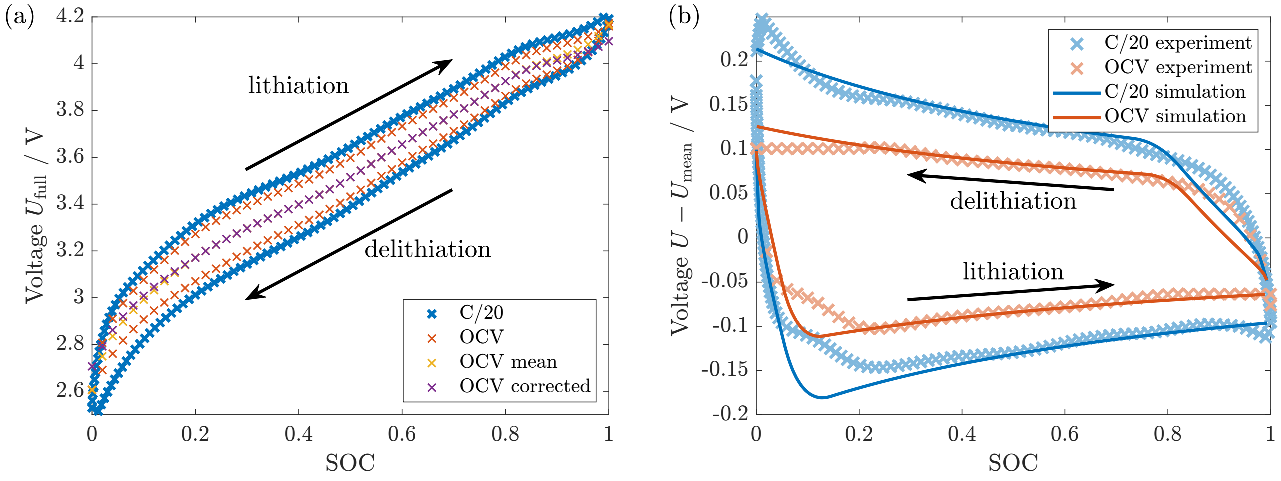

Figure S3. (a) Mean OCV and corrected OCV in comparison to the experimental data [1]. (b) Chemo-mechanical simulation of the voltage hysteresis in comparison to the experimental data related to the corrected OCV curve [1].

SV. COMPARISON WITH GITT MEASUREMENT

Here, we compare our chemo-mechanical core-shell model with the galvanostatic intermittent titration technique (GITT) measurements from Pan et al. [2, 3] for silicon anode half-cells. We use the parameters stated in Table S1 gained in our manuscript from the voltage relaxation measured for silicon anode full cells [1]. Fig. S4(a) reveals a reasonable agreement between the simulated GITT voltage curve and the experimental curve [2]. Fig. S4(b) shows the comparison of our simulation and the experiment for a single GITT pulse. The voltage profiles for the current phase and the relaxation period look similar, while the difference between lithiation voltage and relaxed voltage is smaller for the simulation compared to the data. Particularly, the simulation predicts smaller voltage relaxations for delithiation at high SOC and for lithiation at low SOC values. This deviation results from a slightly too slow voltage transition directly after change of direction predicted by the model compared to the experiment. Additionally, the relaxation appears smaller than it is due to the large slope of the voltage curves in the respective regimes. The deviation of the absolute voltage values is expected as the exact parameters are gained from the relaxation experiment performed with different cells.

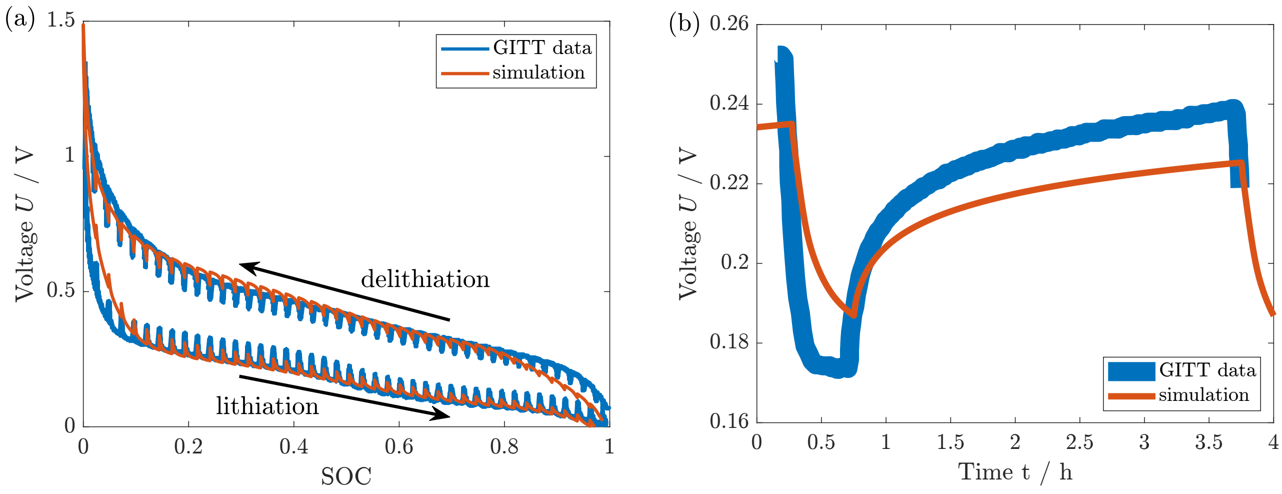

Figure S4. (a) Simulation of GITT procedure with the model and parameters obtained for the long-term relaxation experiment from Ref. [1] in comparison to the experimental data presented in Ref. [2]. (b) Simulation of the voltage transition of a single GITT pulse in comparison to the experimental data presented in Ref. [3].

SVI. C-RATE DEPENDENCE OF REACTION OVERPOTENTIALS

Contrary to our chemo-mechanical model, reaction kinetics are considered in literature as explanation hypothesis for the voltage hysteresis of silicon anodes [4]. For comparison with our model, we evaluate the C-rate dependence of reaction overpotentials in the following.

The relation between the current density j and the reaction overpotential ΔU is commonly described by the Butler-Volmer equation

$$j = -j_0 \left[\exp\left(\frac{\alpha_a F \Delta U}{R_{\text{gas}} T} \right) - \exp\left(-\frac{\alpha_c F \Delta U}{R_{\text{gas}} T} \right) \right] \tag{S37}$$

with the exchange current density j_0 as well as the anodic and cathodic transfer coefficients α_a and α_c. The remaining parameters are the Faraday constant F, the universal gas constant R_{gas} and the temperature T.

Typically for charge-transfer reactions in lithium-ion batteries, the transfer coefficients are assumed to be $\alpha_a = \alpha$ and $\alpha_c = 1 - \alpha$ with $\alpha = 0.5$. Slight deviations of $\alpha = 0.5$ are possible but less common. Values of $\alpha_a, \alpha_c > 1$ are considered to be physically unreasonable.

Figure S5 depicts the C-rate dependence of the reaction overpotentials predicted by the Butler-Volmer equation for different sets of parameters. The exchange current density j_0 is adjusted to meet the OCV at vanishing current and the experimental voltage measured at C/10. The plot shows that typical parameters of $\alpha = 0.5$ can not explain the observed C-rate dependence. In contrast, unreasonable parameter values $\alpha_a > 2$ are necessary to approach the experimental C-rate dependence. Therefore, reaction overpotentials are not able to explain the observed C-rate dependence with reasonable parameter values.

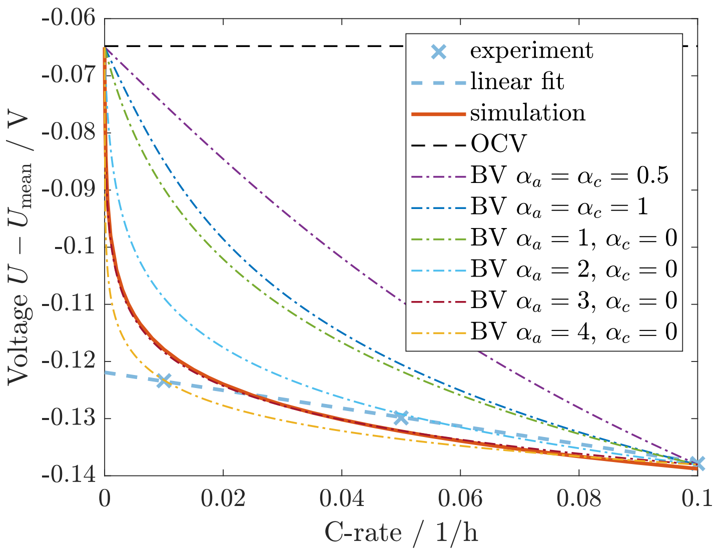

Figure S5. C-rate dependence of Butler-Volmer reaction overpotentials for various reasonable and unreasonable parameters in comparison to experimental data [1].

SVII. VOLTAGE PROFILES OF LITHIATION PULSES

We simulate the voltage profile according to our chemo-mechanical core-shell model during a lithiation after a rest period of 12 h following protocol SIII E. Fig. S6 shows the voltage transition for an interrupted lithiation pulse with C/20 at different SOC values in comparison to a continuous lithiation (dashed lines). The initial voltage difference decreases, and the transition to the lithiation voltage gets slower for higher SOC values. For comparison, we show the experimental result [1] (lighter colors). For the experiment, the initial voltage difference decreases slightly, and the voltage transition shows a slight retardation for higher SOC values. Thus, the general trends of our chemo-mechanical simulation agree with the ones of the experiment. However, all experimental curves show an overshoot instead of a smooth convergence to the lithiation voltage, which is not visible in our simulations.

Such a voltage overshoot during the transition from a rest period to lithiation is measured in Ref. [1] consistently for different C-rates, SOC values, and Si contents in the anode. In terms of mechanics, this overshoot might result from a thixotropic behavior of the shell. Thixotropy means that the viscosity of a material reduces during a deformation period but increases again during a rest period. This increase in viscosity is due to a reorganization of particles or grains inside the material. Assuming the thixotropic behavior of the shell can result in an initially steeper voltage transition and an overshoot analogous to the experimental data shown in Figure S6.

Fig. S7(a) depicts the behavior of an interrupted lithiation pulse for different C-rates after a rest period of 12 h at SOC = 0.5. All curves show a steep slope at the beginning attributed to viscous stress. For $\Delta SOC > 0.01$, it is followed by a slower convergence to the lithiation voltage attributed to elastoplastic behavior. A purely elastic regime is not visible in this scenario, as elastic stress is not significantly relieved during relaxation in contrast to a transition between lithiation and delithiation. The similar slope of the voltage profiles for different C-rates indicates that the voltage transition is dependent on the charge throughput as measured by Wycisk et al. [1] for a blended graphite-silicon anode with 20 % silicon in the low-SOC regime, where silicon is the active component. In contrast, Figure S7(b) reveals that the time dependence of the voltage transition differs strongly for different currents.

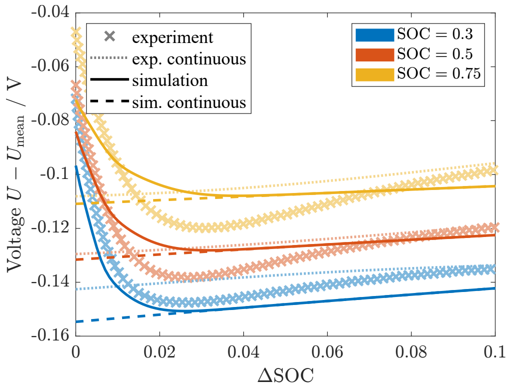

Figure S6. Simulated voltage profile of a lithiation pulse at different SOC after a rest period and the voltage during continuous lithiation depending on the change in SOC in comparison to experimental data [1].

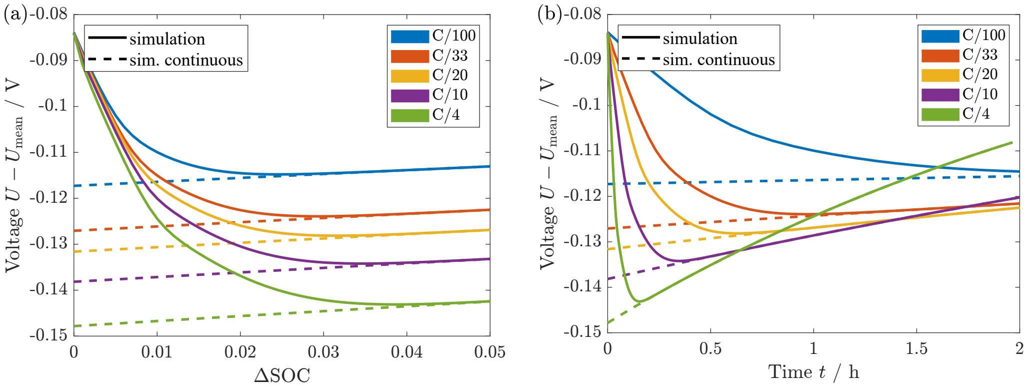

Figure S7. Simulated voltage profile of a lithiation pulse with different C-rates after a rest period and the voltage during continuous lithiation. (a) Voltage profile depending on the change in SOC. (b) Voltage profile depending on the elapsed time.

SVIII. PLETT MODEL

The phenomenological Plett model [5–7] can describe the hysteresis phenomenon and transitions between the voltage curves observed for lithiation and delithiation. According to the Plett model, the measured voltage is the mean open-circuit voltage $U_{\mathrm{mean}}(\mathrm{SOC})$ changed by half of the width of the voltage hysteresis measured between lithiation and delithiation $H(\mathrm{SOC})$ times the hysteresis state $h(\mathrm{SOC})$ defined between -1 and 1 as

$$U = U_{\mathrm{mean}}(\mathrm{SOC}) + H(\mathrm{SOC}) \cdot h(\mathrm{SOC}). \tag{S38}$$

The voltage considering the hysteresis behavior results from the differential equation

$$\frac{dh(\mathrm{SOC})}{d\mathrm{SOC}} = -k(\mathrm{SOC}) \left(1 + \mathrm{sgn}\left(\frac{d\mathrm{SOC}}{dt}\right) h(\mathrm{SOC})\right). \tag{S39}$$

The parameter k determines the slope of the transition between the two hysteresis states, which can in principle depend on the SOC. As a simple relation, we set $k(\mathrm{SOC}) = k_0 = 40$.

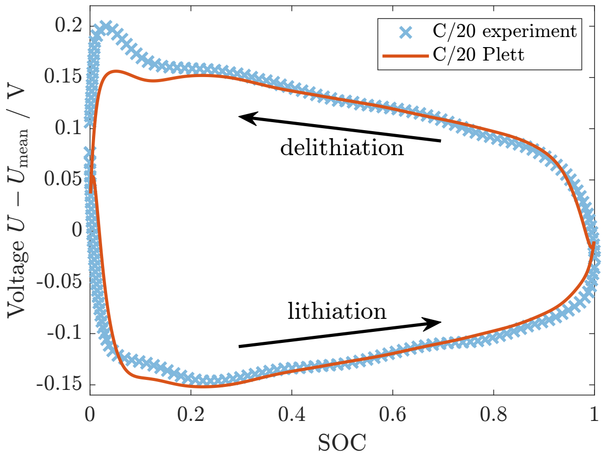

Figure S8. Voltage hysteresis described by the empirical Plett model in comparison to experimental data [1].

SIX. PARAMETERS

Parameter	Value		Reference
**Silicon core**			
Nanoparticle radius R_{core}	50	nm	estimated [2]
Solid state diffusion coefficient in silicon D_{Li}	$1 \cdot 10^{-17}$	$\mathrm{m^2/s}$	[8]
Young's modulus E_{core}	200	GPa	estimated [9, 10]
Poisson's ratio ν_{core}	0.22		[11]
First Lamé constant $\lambda_{\mathrm{core}} = 2G_{\mathrm{core}}\nu_{\mathrm{core}}/\left(1 - 2\nu_{\mathrm{core}}\right)$	64	GPa	calculated
Second Lamé constant $G_{\mathrm{core}} = E_{\mathrm{core}}/2(1 + \nu_{\mathrm{core}})$	82	GPa	calculated
Yield stress $\sigma_{\mathrm{Y,core}}$	3	GPa	fit
Theoretical maximum lithium concentration in Si core $c_{\mathrm{Li,max}}$	$311 \cdot 10^3$	$\mathrm{mol/m^3}$	[12]
Lithium concentration in Si core at SOC = 0	0.1	$c_{\mathrm{Li,max}}$	estimated
Lithium concentration in Si core at SOC = 1	0.9	$c_{\mathrm{Li,max}}$	estimated
Lithium molar volume in Si core v_{Li}	$9 \cdot 10^{-6}$	$\mathrm{m^3/mol}$	[13]
**SEI shell**			
Shell thickness L_{shell}	20	nm	estimated [14]
Young's modulus E_{shell}	100	GPa	estimated [15, 16]
Poisson's ratio ν_{shell}	0.3		[15]
First Lamé constant $\lambda_{\mathrm{shell}} = 2G_{\mathrm{shell}}\nu_{\mathrm{shell}}/\left(1 - 2\nu_{\mathrm{shell}}\right)$	58	GPa	calculated
Second Lamé constant $G_{\mathrm{shell}} = E_{\mathrm{shell}}/2(1 + \nu_{\mathrm{shell}})$	38	GPa	calculated
Yield stress $\sigma_{\mathrm{Y,shell}}$	2.0	GPa	fit
Newtonian viscosity η_{shell}	$135 \cdot 10^{12}$	Pa s	fit
Viscous reference stress σ_{ref}	133	MPa	fit
Viscous time constant τ	$3 \cdot 10^8$	s	fit
**Universal constants and other parameters**			
Temperature T	298	K	
Faraday constant F	96 485	C/mol	
Universal gas constant R_{gas}	8.314	J/(mol K)	

TABLE S1. List of the simulation parameters.

[1] D. Wycisk, G. K. Mertin, M. Oldenburger, O. von Kessel, and A. Latz, Challenges of open-circuit voltage measurements for silicon-containing Li-Ion cells, Journal of Energy Storage **89**, 111617 (2024).

[2] K. Pan, F. Zou, M. Canova, Y. Zhu, and J.-H. Kim, Systematic electrochemical characterizations of Si and SiO anodes for high-capacity Li-Ion batteries, Journal of Power Sources **413**, 20 (2019).

[3] K. Pan, *A Systematic Methodology for Characterization and Prediction of Performance of Si-based Materials for Li-ion Batteries*, Ph.D. thesis, Ohio State University (2020).

[4] V. A. Sethuraman, V. Srinivasan, and J. Newman, Analysis of Electrochemical Lithiation and Delithiation Kinetics in Silicon, Journal of The Electrochemical Society **160**, A394 (2013).

[5] G. L. Plett, Extended Kalman filtering for battery management systems of LiPB-based HEV battery packs, Journal of Power Sources **134**, 262 (2004).

[6] C. P. Graells, M. S. Trimboli, and G. L. Plett, Differential hysteresis models for a silicon-anode Li-ion battery cell, in *2020 IEEE Transportation Electrification Conference & Expo (ITEC)*, Vol. 1 (IEEE, 2020) pp. 175–180.

[7] D. Wycisk, M. Oldenburger, M. G. Stoye, T. Mrkonjic, and A. Latz, Modified Plett-model for modeling voltage hysteresis in lithium-ion cells, Journal of Energy Storage **52**, 105016 (2022).

[8] J. Li, X. Xiao, F. Yang, M. W. Verbrugge, and Y.-T. Cheng, Potentiostatic Intermittent Titration Technique for Electrodes Governed by Diffusion and Interfacial Reaction, The Journal of Physical Chemistry C **116**, 1472 (2012).

[9] M. Nasr Esfahani and B. E. Alaca, A Review on Size-Dependent Mechanical Properties of Nanowires, Advanced Engineering Materials **21**, 1900192 (2019).

[10] D.-M. Tang, C.-L. Ren, M.-S. Wang, X. Wei, N. Kawamoto, C. Liu, Y. Bando, M. Mitome, N. Fukata, and D. Golberg, Mechanical Properties of Si Nanowires as Revealed by in Situ Transmission Electron Microscopy and Molecular Dynamics Simulations, Nano Letters **12**, 1898 (2012).

[11] V. Shenoy, P. Johari, and Y. Qi, Elastic softening of amorphous and crystalline Li–Si Phases with increasing Li concentration: A first-principles study, Journal of Power Sources **195**, 6825 (2010).

[12] A. Verma, A. A. Franco, and P. P. Mukherjee, Mechanistic Elucidation of Si Particle Morphology on Electrode Performance, Journal of The Electrochemical Society **166**, A3852 (2019).

[13] R. Koerver, W. Zhang, L. de Biasi, S. Schweidler, A. O. Kondrakov, S. Kolling, T. Brezesinski, P. Hartmann, W. G. Zeier, and J. Janek, Chemo-mechanical expansion of lithium electrode materials – on the route to mechanically optimized all-solid-state batteries, Energy & Environmental Science **11**, 2142 (2018).

[14] J. Zheng, H. Zheng, R. Wang, L. Ben, W. Lu, L. Chen, L. Chen, and H. Li, 3D visualization of inhomogeneous multi-layered structure and Young's modulus of the solid electrolyte interphase (SEI) on silicon anodes for lithium ion batteries, Phys. Chem. Chem. Phys. **16**, 13229 (2014).

[15] H. Shin, J. Park, S. Han, A. M. Sastry, and W. Lu, Component-/structure-dependent elasticity of solid electrolyte interphase layer in Li-ion batteries: Experimental and computational studies, Journal of Power Sources **277**, 169 (2015).

[16] Y. Chai, W. Jia, Z. Hu, S. Jin, H. Jin, H. Ju, X. Yan, H. Ji, and L.-J. Wan, Monitoring the mechanical properties of the solid electrolyte interphase (SEI) using electrochemical quartz crystal microbalance with dissipation, Chinese Chemical Letters **32**, 1139 (2021).
